# A Novel Stratification Method in Linkage Studies to Address Inter- and Intra-Family Heterogeneity in Autism

**DOI:** 10.1371/journal.pone.0067569

**Published:** 2013-06-26

**Authors:** Zohreh Talebizadeh, Dan E. Arking, Valerie W. Hu

**Affiliations:** 1 Medical Genetics Research, Children’s Mercy Hospitals and Clinics and University of Missouri-Kansas City School of Medicine, Kansas City, Missouri, United States of America; 2 McKusick-Nathans Institute of Genetic Medicine, Johns Hopkins University School of Medicine, Baltimore, Maryland, United States of America; 3 Department of Biochemistry and Molecular Medicine, The George Washington University School of Medicine and Health Sciences, Washington, District of Columbia, United States of America; Sanjay Gandhi Medical Institute, India

## Abstract

Most genome linkage scans for autism spectrum disorders (ASDs) have failed to be replicated. Recently, a new ASD phenotypic sub-classification method was developed which employed cluster analyses of severity scores from the Autism Diagnostic Interview-Revised (ADI-R). Here, we performed linkage analysis for each of the four identified ADI-R stratified subgroups. Additional stratification was also applied to reduce intra-family heterogeneity and to investigate the impact of gender. For the purpose of replication, two independent sets of single nucleotide polymorphism markers for 392 families were used in our study. This deep subject stratification protocol resulted in 16 distinct group-specific datasets for linkage analysis. No locus reached significance for the combined non-stratified cohort. However, study-wide significant (P = 0.02) linkage scores were reached for chromosomes 22q11 (LOD = 4.43) and 13q21 (LOD = 4.37) for two subsets representing the most severely language impaired individuals with ASD. Notably, 13q21 has been previously linked to autism with language impairment, and 22q11 has been separately associated with either autism or language disorders. Linkage analysis on chromosome 5p15 for a combination of two stratified female-containing subgroups demonstrated suggestive linkage (LOD = 3.5), which replicates previous linkage result for female-containing pedigrees. A trend was also found for the association of previously reported 5p14-p15 SNPs in the same female-containing cohort. This study demonstrates a novel and effective method to address the heterogeneity in genetic studies of ASD. Moreover, the linkage results for the stratified subgroups provide evidence at the gene scan level for both inter- and intra-family heterogeneity as well as for gender-specific loci.

## Introduction

Autism is a common early onset neurodevelopmental disorder belonging to a group of conditions known as autism spectrum disorders (ASDs), which include classical autism, pervasive developmental disorder-not otherwise specified and Asperger syndrome [1]. Although there is strong evidence for genetic involvement in susceptibility to ASD [2], the presence of aberrant behaviors across the three core domains of ASD (deficits in communication and social interaction as well as restricted interests and repetitive behaviors) is still the cornerstone for diagnosis. Based on parent interviews by a trained clinician, the Autism Diagnostic Interview-Revised (ADI-R) [1] is widely recognized as one of the gold standard assessment measures for establishing a clinical diagnosis of autism.

It is now generally accepted that multiple genes contribute to the etiology of autism, but the questions of how many susceptibility genes are involved and how they relate to respective subgroups of individuals remain unanswered. To date, several independent genome-wide linkage studies have been performed to investigate the genetic underpinnings of ASD, but with limited success, since the majority of the identified linked regions have not been replicated (see **Table S1** in **File S1** for detail on previously reported linkage).

In the most recent linkage scan studies, the use of genotyping microarray data in international collaborative projects have significantly increased both genome-wide marker coverage and sample sizes in the study cohorts to enhance the chance of finding autism susceptibility loci. In 2007, a genotyping study that interrogated ∼10,000 SNPs in more than 1,000 families in the phase one Autism Genome Project (AGP) found no genome-wide significant linkage peaks, but detected suggestive linkage at 11p and 15q chromosomal regions [3]. Partitioning families based on the affected proband’s gender (i.e., male-only and female-containing pedigrees) provided evidence for gender-specific autism susceptibility loci. Despite an improvement in linkage data following the implementation of gender stratifications, none of the results reached a genome-wide statistically significant level.

In the second largest autism linkage study reported in 2009, more than 800 families and 16,000 rigorously filtered SNPs were included [4]. The two aforementioned suggestive loci identified in the AGP study were not seen in this autism cohort and the top linkage signals were detected for two new loci (LOD = 2.94 at 6q and LOD = 3.81 at 20p). The failure to replicate linked loci, even with a large cohort size that was predicted to have enough power for detecting autism-linked loci [3], further underscores the fact that increasing sample size is necessary but not sufficient to tackle the major challenge posed by the extensive heterogeneity in this population.

The heterogeneous phenotype of autism suggests the need to employ strategies to identify homogeneous groups of subjects with common or more similar features. There have been attempts at phenotypic stratification that focus on different ADI-R criteria, such as language related phenotypes, by use of scores on ADI-R items corresponding to phrase speech delay [5], [6], age at first words [7]–[10], and reading impairment [11], while other studies differentiate subgroups using narrow and broad ASD diagnoses [7], [12]–[15] and gender [7], [16]–[18]. In many cases, studies using stratification to reduce heterogeneity have led to linkage signals on loci not previously identified as well as increased signals despite reductions in sample sizes. However, many of these studies stratified subjects based on severity along a single domain, such as language impairment or nonverbal communication, while individuals with ASD manifest deficiencies across a broad range of behaviors.

Recently, Hu and Steinberg [19], identified four subgroups of autistic individuals by evaluating ADI-R scores across a broad range of symptoms using multiple clustering methods. Subsequent expression profiling of lymphoblastoid cell lines derived from individuals within three of the four phenotypic subgroups by DNA microarray analyses revealed both overlapping as well as unique subtype-dependent genes that were differentially expressed relative to control samples [20]. The gene expression study suggested that the symptomatic subtypes derived from the ADI-R cluster analyses may represent distinct biological phenotypes [20]. Recently, similar application of phenotypic clusters to re-analyze data from a published genome-wide association study (GWAS) [21] improved the ability to identify statistically significant novel ASD subtype-associated SNPs [22]. In the present study, the same four ADI-R subject clusters were used in linkage analysis to investigate whether this subject stratification method also improves linkage analyses of ASD.

## Materials and Methods

### Genome-wide SNP Data and ADI-R Subtypes

Two independent datasets of single nucleotide polymorphism (SNP) were utilized to perform the linkage analysis. SNP dataset-1 contains data on approximately 8,000 markers throughout the genome derived from the Affymetrix 10 K SNP array, generated from >1000 families in the phase one AGP [3]. Marker exclusion criteria included minor allele frequency <0.05 (removed 1,242 SNPs), high rate of missing genotypes (removed 1,112 SNPs) [3], and deviation from Hardy-Weinberg Equilibrium (removed 207 SNPs). SNP dataset-2 contains genome-wide markers (16,303 autosomal and 670 X-linked) that were used in a more recent linkage study involving >800 families [4]. The latter dataset was created by combining high quality SNPs from Affymetrix 5.0 and 500 K array platforms, as previously described [4]. Quality control filtration applied to this SNP dataset included >99.5% concordance of genotyping obtained by two array platforms and ≤1 Mendelian error [4].

Our subject inclusion criteria were the availability of both the ADI-R related cluster assignment of the probands [19] and the two SNP datasets [3], [4]. A total of 392 multiplex families from the Autism Genetic Resource Exchange (AGRE) met the inclusion criteria and were used for our linkage analysis. The self-reported race of these subjects includes 76% white, 14% unknown, 5% Asian, 2% mixed, 2% African American, and 1% native Hawaiian or other Pacific Islander. The prevalence of the common race (i.e., white) in each subgroups are listed in **Table S2** in **File S1**. Both parents were mostly genotyped which minimizes the impact of ethnic specific allele frequencies on linkage analysis.

### Sub-phenotype Analysis

Phenotypic subtyping of the probands was assigned using previously performed ADI-R cluster analyses methods [19]. See **File S1** for detail on the clustering method. In this study, these four ADI-R subgroups are referred to as the following: (g1) severe, with language impairment, (g2) mild, with lower symptom severity across all items, (g3) moderate, with notable savant skills, and (g4) intermediate phenotype.

**Figure 1:**
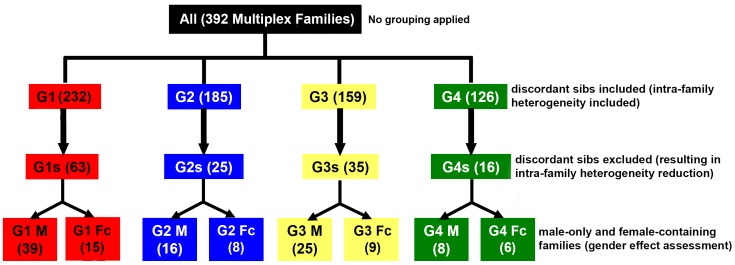
Description of the stratification protocol used in this study. The 4 original sub-phenotypes (denoted by four different colors) were further stratified by removal of families containing affected siblings of another sub-phenotype to yield the Gs level subgroups. These subgroups were further divided according to male only or female-containing pedigrees. Due to intra-family heterogeneity in multiplex cases, some families were included in more than one stratified group. Therefore, the sum of individual and family numbers in subgroups exceeds the numbers listed for the original combined cohort (ALL).

The affected subject’s ADI-R sub-phenotype (i.e., g1, g2, g3, or g4) was used to create group-specific SNP datasets using a three-step stratification process as shown in **Figure 1** [Step 1] G level: AGRE multiplex families having at least one autistic individual (proband) belonging to a specific ADI-R sub-phenotypic cluster were sorted into the relevant phenotypic group (i.e., G1, G2, G3, or G4). Therefore, the G level grouping of pedigrees is based on the proband identified with that specific subtype of ASD, and all affected siblings were included regardless of their ASD subtype. [Step 2] Gs level: affected siblings that were not in the same phenotypic subgroup (i.e., discordant siblings) were removed from the G level groups to reduce intra-family heterogeneity, resulting in an additional level of subject stratification (i.e., G1s, G2s, G3s, and G4s). For example, the G1s group contains only those multiplex families in which all affected siblings fall into the ADI-R related g1 category. [Step 3] Gender-specific level: to assess gender effect, the analysis was also done based on the concordant affected individual’s gender [i.e., male only (GM) and female-containing (GFc) pedigrees], allowing further reduction in heterogeneity.

Initially, only subjects with a strict classification of autism by AGRE were included (n1) in our analysis, and broad spectrum subjects were removed. Upon completion of our initial linkage scans, broad spectrum subjects were then added (n2) to each subgroup based on their ADI-R-determined sub-phenotypes [19]. This step resulted in the expansion of sample sizes in all subgroups, except the female-containing sets. Linkage analysis was performed on the expanded stratified pedigree datasets to assess the impact of increasing sample size on linkage results. The numbers of multiplex families for each subgroup that resulted from the aforementioned subject sub-phenotyping methods are shown in **Table S2** in **File S1**. Also shown is the number of families in the original group of combined cases (referred to as “ALL”).

### Linkage Analysis

See **Figure S2** in **File S1** for detail on linkage analysis and permutation. Since the second SNP dataset has undergone a more rigorous filtration, the reported LOD scores in this study are based on the values obtained using this dataset. The AGP SNP dataset [3] and ADI-R scoresheets [19] were downloaded from the AGRE website. The second SNP dataset was obtained from the Weiss *et al*. paper [4].

#### Ethics Statement

N/A.

## Results

### Stratification Pipeline and Research Plan

Our stratification workflow and the resulting 16 subgroups for the linkage study are illustrated in **Figure 1**. Because of intra-family sub-phenotypic heterogeneity, some pedigrees overlap at the G level, as shown in **Table S3** in **File S1**. The applied multi-step stratification process provided a pipeline to further filter the original heterogeneous ASD pedigree data file (ALL) to more homogeneous datasets by first using ADI-R cluster analysis, followed by removal of sub-phenotypically discordant siblings, and finally by separation of male-only and female-containing pedigrees.

To assess whether genotyping quality or artifacts contributes to our results, linkage analysis was performed at the discovery and validation phases, using two independent SNP datasets. We first ran linkage analyses using SNP dataset-1 (i.e., discovery phase). Next, the replication of suggestive linkage results was assessed by repeating genome-wide linkage, for the same subgroups, using SNP dataset-2 (i.e., validation phase). The reported LOD scores represent values that have been generated by the second SNP dataset because the second SNP dataset has been subject to a more rigorous quality control filtration.

To assess the impact of increasing sample size on linkage results, we added subjects described as “broad spectrum” by AGRE to the initial cohort which included only subjects with a strict diagnosis of autism [denoted as n1 in **Table S2** in **File S1**]. This addition of broad spectrum subjects increased sample sizes in all groups except female-containing subsets [denoted as n2 in **Table S2** in **File S1**].

### Linkage Analysis Results

Genome-wide linkage analyses were performed, separately, on n1 and n2 subject cohorts. After applying subject stratification, the LOD scores were improved in many regions compared to the combined (ALL) group, and new subgroup-specific suggestive linkage regions were detected, despite the reduced sample size in each subgroup. The highest LOD score obtained for the ALL group in the n1 cohort (n = 337 families) was 1.98 for chromosome 10q22 (data not shown). After increasing the sample size to 392 families (i.e., the n2 cohort), the positive LOD score at the 10q22 locus for ALL group decreased to 1.61. However, as shown in **Table 1**, LOD scores for two loci (13q21 and 12q21) exceeded 3.0 in the n1 cohort, for the G1 and G4s subgroups, respectively. These linkage scores were both improved after adding new subjects in the validation phase, reaching 4.37 and 3.56 LODs, respectively. Furthermore, two positive peaks detected in the n1 cohort for G1s (22q11, LOD = 1.41) and G4s (11p15, LOD = 2.83), exceeded a LOD of 3 in the n2 cohort (LODs = 4.43 and 3.13, respectively). Simulation analyses (using 100 simulated files containing randomized cohorts) were used to determine the significance of the observed LOD score, accounting for the multiple testing due to subgroup analyses. These applied permutation tests (described in the methods section and shown in **Tables S9A** in **File S3** and **S9B** in **File S4** demonstrated that the top two linkage scores obtained for the G1 and G1s subsets (LOD>4 in **Table 1**) at 13q21 and 22q11, respectively, reached study-wide significance (p = 0.02).

**Table 1:** Improvement of maximum LOD scores in subgroups with addition of new families.

		LOD score [p value] (# of multiplex families)	
Locus	Subgroup	Cohort 1 (n1)	Cohort 2 (n2)
13q21^1^	G1	3.87 [0.00001] (194)	4.37 [0.00001] (232)
22q11	G1s	1.41 [0.005] (41)	4.43 [0.00000] (63)
11p15^1^	G4s	2.83 [0.00014] (13)	3.13 [0.00007] (16)
12q21^1^	G4s	3.25 [0.00005] (13)	3.56 [0.00003] (16)

1 Previously reported linked region (see **Table S1** in **File S1** for references).

**Table 2** compares these four max LOD scores with the results obtained at the same locus for the undivided “ALL” group as well as the scores for the stratified subgroups [see **Table S4** in **File S2** for the LOD scores for each of the stratified groups]. Such a side-by-side comparison demonstrates that the highest linkage scores may be achieved at different levels of stratification (e.g., G or Gs levels). For example, locus 13q21 is potentially a shared region (LOD = 4.37) for all affected siblings in G1 pedigrees (232 multiplex families) regardless of the sub-phenotype of siblings. After excluding discordant siblings, 169 of 232 G1 pedigrees (73%) are no longer multiplex and thus cannot contribute to linkage. This substantial reduction of the number of pedigrees (from 232 to 63) causes loss of linkage peak for this region in the G1s group (LOD = 0), demonstrating a pattern best fitting with intra-family shared regions. On the other hand, removal of discordant sub-phenotypes within pedigrees, to generate Gs level families, resulted in significantly improved LOD scores for the three remaining loci listed in **Table 1**. These results demonstrate that intra-family phenotypic heterogeneity may also confound linkage studies.

**Table 2:** Loci with highest LOD scores for a given subtype.

		LOD score [p value] per group (# of multiplex families)				
**GROUP 1**						
**Locus**	**SNP**	**ALL (392)**	**G1 (232)**	**G1s (63)**	**G1M (39)**	**G1Fc (15)**
13q21^1^	rs4142274	1.79 [0.002]	a **4.37 [0.00001]** *	0.0 [0.03]	0.0 [0.5]	0 [0.3]
22q11	rs2283792	1.27 [0.008]	1.53 [0.004]	b **4.43 [0.00000]** *	1.63 [0.003]	2.54 [0.0003]
**GROUP 4**						
**Locus**	**SNP**	**ALL (392)**	**G4 (126)**	**G4s (16)**	**G4M (8)**	**G4Fc (6)**
11p15^1^	rs2028608	0.42 [0.08]	0.15 [0.2]	b **3.13 [0.00007]**	1.94 [0.0014]	0.89 [0.02]
12q21^1^	rs10735989	0.06 [0.3]	0.59 [0.05]	b **3.56 [0.00003]**	1.7 [0.003]	1.55 [0.004]

The highest LOD scores (shown in bold font), were obtained after including additional families (i.e., n2), as described in **Table S2** in **File S1**.

1 Previously reported linked region (see **Table S1** in **File S1** for references).

* According to permutation tests reached a study-wide significant (i.e., p = 0.02, see **Table S9A** in **File S3**); G1 = 87% white, G1s = 98% white.

a An example of loci with highest LOD scores for the first level of subgrouping (intra-family heterogeneity included). This is potentially a shared linked region for all affected siblings in a pedigree regardless of concordance status, for a given subtype (i.e., G1).

b Loci with highest LOD scores when only group-specific concordant autistic subjects were maintained (intra-family heterogeneity reduced). It is potentially a linked region only for concordant siblings in a given subtype (i.e., G1s and G4s).

Despite the observed differences in linked regions among these ADI-R subtypes, several overlapping linkage signals were also seen for different subgroups. For example, two separate loci (5p15 and 22q11) with positive LOD scores were shared by G1Fc and G2Fc subgroups (**Table 3**). To assess the validity of such shared loci, a new combined genotype dataset that included both relevant subgroups was compiled. Computed LOD scores for four such combined datasets and the original single subgroup scores are shown in **Table 3**. In all four cases, the shared linkage result was improved and reached a suggestive linkage score (LOD>3) in the combined datasets. Such an additive effect and particularly reaching a LOD score of 3 upon merging two groups was not seen for all the loci with a similar pattern in non-combined groups. Thus, we speculate that the examples shown in **Table 3** may potentially represent shared linkage regions between the two merged groups. This conclusion should be taken with caution because the merged LOD scores did not pass permutation corrections and need further confirmation.

**Table 3:** Linkage data obtained for four overlapping regions, between two different subgroups.

Overlapped region	SNP	Subtype [LOD score (p value)] # of multiplex families			
5p15^1^	rs4701995	ALL [0.97 (0.02)] 392	G1Fc [1.76 (0.002)] 15	G2Fc [1.94 (0.0014)] 8	G1Fc & G2Fc [3.50 (0.00003)] 23
22q11	rs2283792	ALL [1.27 (0.008)] 392	G1Fc [2.54 (0.0003)] 15	G2Fc [0.69 (0.04)] 8	G1Fc & G2Fc [3.23 (0.00006)] 23
15q25	rs2654209	ALL [1.03 (0.015)] 392	G1M [2.52 (0.0003)] 39	G3M [0.79 (0.03)] 25	G1M & G3M [3.12 (0.00008)] 89
17q11^1^	rs11658900	ALL [2.94^a^ (0.00012)] 392	G3 [1.58 (0.003)] 159	G4 [1.86 (0.002)] 126	G3 & G4 [3.33^a^ (0.00004)] 249

Calculated LOD scores were improved after combining the two respective subject groups. It further validates the original computed LOD scores and serves as a partial replication of our linkage results.

1 Previously reported linked region (see **Table S1** in **File S1** for references).

a A positive LOD score of 2.94 was obtained when no stratification was applied to 392 families (i.e., ALL). The linkage results shown here for the SNP rs11658900 suggest that the subgroups G3 and G4 are the strongest contributors to the original LOD score in the unstratified cohort (ALL). Therefore, combining G3 and G4 data resulted in an improvement in the LOD score relative to ALL with fewer families (i.e., 249).

**Figure 2:**
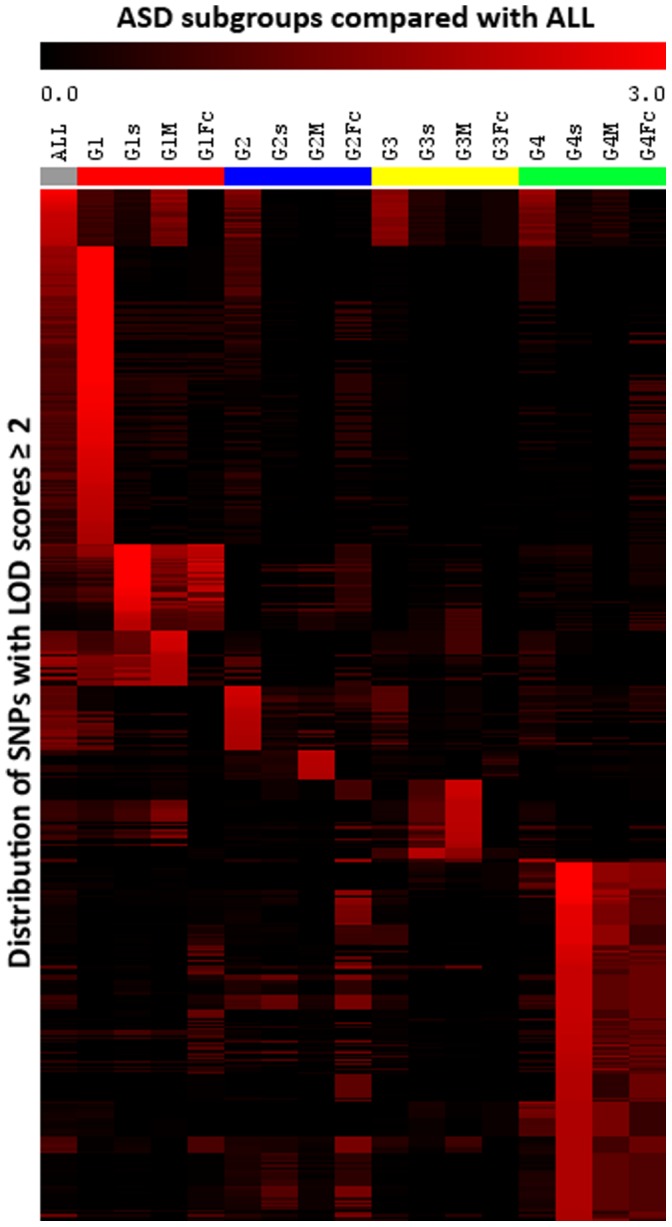
Heat map of LOD scores. A graphical representation (heat map) of the LOD score data (cut-off ≥2.0) was generated to visually demonstrate the computed linkage scores for each subgroup in a hierarchy. The heat map compares LOD score patterns for the 16 subgroups. As expected, there were more similarities within each ADI-R group (e.g., G1, G1s, G1M, and G1Fc) than between two different ADI-R groups. Each horizontal band represents a SNP while each column represents a stratified subgroup, with the exception of the first column which represents the combined (ALL) cohort. **Table S4** in **File S2** lists the SNPs and LOD scores contributing to the identified segregation patterns by subgroups (i.e., hot spots). The corresponding genomic positions of the SNPs contributed to the heat map (Y-axis) are listed in **Table S4** in **File S2**. The heat map was generated using MeV software [41].

**Figure 2** provides a visual representation of the overall distribution of linked loci with LOD scores ≥ 2 for each of the stratified groups [listed in **Table S4** in **File S2**]. In this figure, LOD scores are displayed as a linkage heat map (using a supervised method) which shows improved linkage in at least one of the stratified subgroups relative to the undivided ALL group (see **File S1** for detail on method). Chromosomal locations of the positive linked loci and their associated genes are summarized by subgroup in **Table S5** in **File S1**. What is clear from this visual map of genome-wide LOD scores across the stratified subgroups is that reduction of phenotypic heterogeneity on the basis of cluster analyses of severity scores across a broad spectrum of ASD symptoms and behaviors greatly improves the ability to identify genetic linkage for specific sub-phenotypes of ASD. Unsupervised hierarchical clustering analysis and principal components analysis of this data further corroborate sub-phenotype dependent linkage results (**Figure S1** in **File S1**).

### Comparison of Linked Regions with SNPs Identified by Association Analyses

In previous GWAS studies, the most significant associations for autism have been reported for the SNPs at 5p14 (rs10513025) and 5p15 (rs4307059) [4], [21]. In our study, the G1.2Fc group provided suggestive evidence for linkage to chromosome 5p15 (LOD = 3.5 as listed in **Table 3**). To evaluate if the affected subjects in this combined female-containing group also show evidence of associations to the previously reported SNPs at this chromosomal region, TDT association was performed for the combined G1Fc and G2Fc (G1.2Fc) subjects (23 cases). Nominally significant association was seen for the rs10513025 and rs4307059 SNPs in this subset [**Table S6** in **File S1**]. However, no associations were seen for either SNP when a total of 166 autism cases from all female-containing pedigrees (i.e., without ADI-R stratification) was analyzed (see **File S1** for detail on TDT association method and result).

## Discussion

Disparity in linkage results for autism highlights the degree of genetic heterogeneity both within and among families. Studies of population isolates such as the Finnish [23], the Chinese Han [24], [25], and extended pedigrees of very large families [26] have provided one approach to deal with the clinical heterogeneity in genetic studies, including linkage analysis. However, it remains to be determined how to address heterogeneity in the very well characterized and highly studied autism datasets such as those collected by the AGRE and the AGP that do not fit the isolated populations or extended pedigree scenarios.

To address this critical gap, we reanalyzed previously generated SNP data available from 392 AGRE families, a subset of samples included in both the first phase of the AGP [3] and the Weiss *et al.* study [4] using a multi-step stratification pipeline. The employed stratification method substantially improved linkage results for the more homogeneous subgroups over the original non-stratified group (**Table 2**). Given the samples sizes and multiple testing involved in genome-wide linkage analyses, rigorous simulation analyses were conducted to assess how often a linkage statistic is achieved by chance. It is notable that two of the generated LOD scores for our subgroups (4.43 at 22q11 and 4.37 at 13q21) exceeded the study-wide significance level (P = 0.02), as indicated by the simulation analyses.

From the present study it appears that subsets representing intermediate phenotypes (i.e., G4, G4s, G4M, and G4Fc) are more likely to consist of multi-ethnicity groups, compared with the most severely language impaired subsets (i.e., G1, G1s, G1M, and G1Fc), as shown in **Table S2** in **File S1**. Larger sample sizes, including sufficient number of subjects from different ethnic backgrounds, are required to assess if there exists ethnicity-related variations in the prevalence of the ADI-R subtypes in autistic populations.

### Biological Implications of Most Significant Genome-wide Linkage Results

The genes residing in the linkage intervals may provide some insight into the biology of ASD. The 13q21 region has been previously linked with autistic subjects ascertained for language impairment [11]. The responsible gene(s) for the combined phenotypes has not been yet identified but this region harbors potential candidate genes such as *DIAPH3* with suggested connections to both autism and language impairment. *DIAPH3*, an auditory neuropathy gene whereby affected subjects show impairment of speech perception [27], has been recently reported as an autism risk gene at 13q21 [28]. It has been suggested that *DIAPH3* might be involved in synaptic activity and function downstream of *SHANK3* (chromosome 22q13) [28], a well-documented autism susceptibility gene [29]. The role of *SHANK3* in language development has also been suggested by its implication in cases with a severe speech and language delay [30], [31].

Several lines of evidence have already documented associations of chromosome 22q11 with language related disorders [32]–[35]. The importance of this region in autism has been recently highlighted by the identification of two autism candidate genes, *TBX1* [36] and *GNB1L* [37]. Gene dosage evaluation in a mouse model of 22q11 deletion/DiGeorge syndrome has shown that disruption of genes other than Tbx1 may be potential contributors for developmental disorders including autism associated with this syndrome [38]. *COMT*, one of the autism susceptibility genes in this chromosomal region has also been investigated in correlation with language production and semantic verbal fluency [39].

Given that the 13q21 and 22q11 regions both show the highest LOD scores for the subtypes of ASD with severe language impairment (i.e., G1 and G1s, respectively), the above-mentioned studies and our current linkage findings suggest that further evaluation of genes within these regions is warranted, especially candidate genes (e.g., *DIAPH3*, *SHANK3*, and *COMT*) for ASD individuals with this language-impaired phenotype. See **Tables S5, S7, S8** in **File S1** for more discussion on potential candidate genes in the linkage intervals.

### Study-wide Significant Linkage Results

In the two previous large genome-wide linkage studies [3], [4] more than 1000 and 800 multiplex families were genotyped, respectively. No significant linkage was reached in the first study and one genome-wide linkage signal (LOD = 3.81 at 20q) was found for the latter study by analyzing 800 families. In the present study, we reanalyzed a subset of families (i.e., AGRE families stratified by cluster analyses of ADI-R scores, n = 392) from these two large genome projects. While several linkage signals exceeded the conventional cut-off of 3 (e.g., LOD = 3.56 for G4s with 16 families), study-wide significant linkage (accounting for subgroup analyses) was reached (P = 0.02) for G1 (LOD = 4.37 at 13q21) and G1s (LOD = 4.43 at 22q11) with only 232 and 63 families, a small fraction of what was included in the original projects. Thus, our linkage analysis reveals sub-phenotype dependent loci that otherwise would not have been detected in the undivided sample. It is unlikely that ethnicity would have impacted these family-based linkage results, inasmuch as both G1 and G1s subsets mainly consisted of one race, as shown in **Table S2** in **File S1** (i.e., 83% and 97%, respectively).

In our study, only the G1 and G1s subtypes showed significant linkage to 13q22 and 22q11, respectively. The location of 13q22 linked region is very close to the previously reported region by Bartlett *et al*. [11] in a study of families with reading impairment and ASD diagnosis. The ADI-R g1 subjects in our study represent autistic individuals with severe language impairment. Therefore, we conclude that linkage to 13q21 in G1 is a replication of previous linkage reports, while the 22q11 linked region found in G1s may represent a novel autism locus related to language impairment. This novel linked locus connects the findings for autism and language disorders that have been previously documented for this chromosomal region.

### Inter and Intra Family Heterogeneity

Heterogeneity in ASD is also reflected at the family level. In multiplex families, autistic symptoms may vary among affected siblings. To explore the impact of this layer of heterogeneity on linkage analyses, we adopted a multi-step subject stratification approach, denoted by the G and Gs annotation, wherein intra-family phenotypic heterogeneity was included or reduced, respectively. The linkage data obtained by this stratification method supports the idea that some loci might be common in all affected siblings within a family, as shown by loci producing highest linkage peaks at the G level. On the other hand, some loci exhibited higher LOD scores after reducing intra-family heterogeneity, i.e., at the Gs level (see **Table 2**). Thus, these loci may harbor risk variants only for concordant siblings. As expected, some loci also had the highest linkage scores when gender was taken into account (**Table 3**). These deeply stratified analyses show that the complexity of ASD requires strategies both at the research design and data analysis levels to address multiple sources of heterogeneity.

As observed with gene expression profiling [20] and GWAS [22] studies of ASD subgroups using the same ADI-R-driven sub-phenotyping protocol [19], we also found a number of loci potentially shared between two subtypes in our linkage analyses. The increase or maintenance of suggestive linkage scores with combined datasets of subgroups exhibiting the shared loci (**Table 3**) provides further support for the validity of these linkage data as well as partial replication of the identified loci. Furthermore, partial replication and validation of the identified linked loci were shown by assessing linkage using two independent SNP datasets and improvement of linkage after sample size expansions within the ADI-R subgroups (**Table 1**).

### Potential Relevance of Suggestive Linked Regions to Autism

A suggestive linkage peak at 5p15 was found for the G1.2Fc combined group (LOD = 3.5, p = 0.00003, 23 families). This linkage score did not pass the study-wide significant estimated by permutation tests. However, this suggestive linkage is in agreement with the AGP report where linkage to 5p14.33 was also detected for female-containing families. This concordant finding further emphasizes that female-containing families might be more informative for linkage [3]. The importance of this chromosomal band has been further highlighted by two genome-wide GWAS reports that identified 5p14 and 5p15 as the most significant associated loci for autism. More recently, a novel mechanistic explanation was discovered for autism based on a noncoding RNA at 5p14 which was antisense to the *MSN* gene on chromosome X [40].

Despite small sample sizes, we also found a suggestive association with the G1.2Fc subjects for both of the previously reported SNPs on chromosome 5p. Such a positive trend for association was not detected when assessing all female-containing families, further demonstrating the positive impact of our stratification approach. Together with these recent linkage, GWAS, and noncoding RNA studies, the suggestive linkage and TDT findings in our G1.2Fc group suggest that studying pedigrees in this ASD subset may provide a greater chance of revealing other relevant information in the integrated model proposed by Kerin *et al* [40] for the role that 5p14-p15 region plays in the etiology of autism. The discussion of chromosome 5p findings, exemplifies that how the multi-step integrated approach presented in the current study (i.e., combining phenotypic classification with linkage and association studies) can contribute to the autism field by connecting relevant pieces and identifying susceptible subsets (i.e., G1.2Fc) that may further strengthen previous findings.

### Concluding Remarks

Our study demonstrates a novel and powerful stratification method to address the heterogeneity in autism spectrum disorders within and among families. Herein, we used ADI-R clustering subtyping for subject classifications to test the validity of our multi-step stratification strategy. ADI-R clustering is only one way of stratifying ASD subjects. Similarly, other ASD stratification measurements can be used when employing the present deep stratification method. Such multi-faceted methods (i.e., combining ASD subject classification and family stratification) can be also applied to all genomic studies to improve the likelihood of uncovering previously undetected genetic factors masked by clinical heterogeneity. The number of families examined to identify suggestive linkage regions in the subgroups is considerably fewer than the total number of families in the undivided group. These findings thus illustrate the added likelihood to detect significant linkage when the heterogeneity of the ASD population is reduced by sample stratification. Finally, our present study provides evidence at the linkage level for both inter- and intra-family heterogeneity, reflecting both shared and distinct genetic makeup in the autism population.

## Supporting Information

File S1 This file contains **Figure S1**, Hierarchical clustering and principal components analyses, **Figure S2**, Workflow describing the applied permutation analysis, **Table S1**, A summary of previously reported linkage results for autism, **Table S2**, The number of multiplex families, in each subgroup, without (n1) and with (n2) BroadSpectrum subjects, **Table S3**, Overlap between the subgroups at the G level, **Table S5**, Chromosomal locations of the positive linked loci (LOD≥2) and their associated genes per subgroups, **Table S6**, TDT result for two previously associated SNPs at chromosome 5p, **Table S7**, List of the genes associated with the SNPs with the highest LOD scores in 13q21 (G1 group), and **Table S8**, List of the genes associated with the SNPs with the highest LOD scores in 22q11 (G1s group).
(PDF)

File S2 This file contains **Table S4**, The SNPs and corresponding LOD scores ≥2.0 across all subgroups.
(XLSX)

File S3 This file contains **Table S9A**, Simulation data (17 groups).
(XLSX)

File S4 This file contains **Table S9B**, Simulation data (20 groups).
(XLSX)
