# Supplementary material for: A Novel Stratification Method in Linkage Studies to Address Inter- and Intra-Family Heterogeneity in Autism
Source: PLoS One. 2013 Jun 26;8(6):e67569. doi: 10.1371/journal.pone.0067569 (PMC3694043; doi:10.1371/journal.pone.0067569)
Supplement: File S1 — This file contains Figure S1, Hierarchical clustering and principal components analyses, Figure S2, Workflow describing the applied permutation analysis, Table S1, A summary of previously reported linkage results for autism, Table S2, The number of multiplex families, in each subgroup, without (n1) and with (n2) BroadSpectrum subjects, Table S3, Overlap between the subgroups at the G level, Table S5, Chromosomal locations of the positive linked loci (LOD≥2) and their associated genes per subgroups, Table S6, TDT result for two previously associated SNPs at chromosome 5p, Table S7, List of the genes associated with the SNPs with the highest LOD scores in 13q21 (G1 group), and Table S8, List of the genes associated with the SNPs with the highest LOD scores in 22q11 (G1s group). (PDF) [file pone.0067569.s001.pdf]

A Novel Stratification Method in Linkage Studies to Address Inter- and Intra-Family Heterogeneity in Autism

INTRODUCTION-Supplemental information

Previously Reported Linkage Studies on ASD

An overview of the linked regions can be found in review articles by Freitag *et al*. [1], Abrahams *et al*. [2], Weiss *et al*. [3] as well as Craddock *et al*. [4] which provides a broader review of linkage findings related to psychiatric disorders. A synopsis of the previously reported genome-wide linkage studies clearly shows the need for subject stratification which has been explored in more recent reports. A summary of previously reported linkage studies on ASD is listed in **Table S1.**

Overall, applications of multi-gene models and genome-wide linkage studies have shown several linked loci, but with a wide variance in results. A number of proposed loci harbor promising autism candidate genes; yet subsequent studies have not confirmed their potential role in autism. In several instances, an original suggestive linkage locus even disappeared after sample size expansion. In 1998, the result of a full genome screen from 99 families was reported by the International Molecular Genetic Study of Autism Consortium (IMGSAC) [5]. Several regions showed suggestive linkage and the most significant susceptibility regions were identified on chromosomes 7q and 16p with an maximum multi-point LOD score (MLS) of 3.55 and 1.97, respectively. In 2001, the IMGSAC added additional families and markers, expanding their earlier linkage study to 152 affected sib-pairs [6]. Although the scores on chromosomes 7 and 16 showed an increase when a larger population was analyzed, the previously reported linkage scores for other chromosomes were diminished, despite increasing sample size [6].

In 2001, Liu *et al*. [7] genotyped 335 microsatellite markers in 110 multiplex families with autism from the Autism Genetic Resource Exchange (AGRE), resulting in several new suggestive linkage regions. Yonan *et al*. [8] reported a follow-up genome-wide screen using 345 AGRE families, a sample size that was three times greater than the previous study conducted by the same group [7]. When the sample size was increased to 345 families some scores were improved, while others decreased in comparison to the earlier study. For example, the scores for regions on chromosomes 19 and X were respectively decreased from 3.36 and 2.27 in 110 families to 0.69 and 1.78 in 345 families [7,8]. Such examples of decreased LOD scores with larger sample sizes illustrate some of the problems associated with replicating linkage data and demonstrate that a larger sample size alone does not necessarily translate into improved statistical outcomes. The key questions for genetic analyses are: (i) how many of these loci represent a true susceptibility region and (ii) given the phenotypic heterogeneity among cases, how can the identified loci be best associated with the respective autistic subjects?

Table S1. A summary of previously reported linkage results for autism. Only loci that generated the highest linkage scores are included.

Reference	Phenotypic Criteria	Locus	Statistical Value	Cohort
[5]	ASD diagnosis	7q32.1-q34	multipoint MLS = 3.55	56 UK affected sib pair families
		7q32.1-q34	multipoint MLS = 2.53	87 affected sib pair families
		16p13.2-p13.13	multipoint MLS = 1.97	56 UK affected sib pair families
		16p13.2-p13.13	multipoint MLS = 1.51	87 affected sib pair families
		4p16.2-16.3	multipoint MLS = 1.55	87 affected sib pair families
		4p16.2-16.3	multipoint MLS = 1.1	56 UK affected sib pair families
[9]	ASD diagnosis	13q22	multipoint MMLS/het = 3.0; rec model	75 affected sib-pairs
		13q12	multipoint MMLS/het = 2.3; rec model	75 affected sib-pairs
		7q21	multipoint MMLS/het = 2.2; rec model	75 affected sib-pairs
[10]	ASD diagnosis	1p13.2	MLS = 2.15	139 multiplex families
	ASD diagnosis	17p13.2	MLS = 1.21	139 multiplex families
[11]	ASD diagnosis	6q16.3	multipoint MLS = 2.23 (P = 0.0013)	51 multiplex families
		6q16.3	2 point MLS = 1.02 (P = 0.0149)	51 multiplex families
		18q21.33	2 point MLS = 1.47 (P = 0.0046)	51 multiplex families
		19p13.12	2 point MLS = 1.17 (P = 0.0102)	51 multiplex families
[7]	Broad ASD diagnosis	5p13-5p14	MLS = 2.5 (p = 0.003)	110 AGRE families
	Broad ASD diagnosis	Xq25	X-MLS = 2.56 (p = 0.0023)	110 AGRE families
	Broad ASD diagnosis	19p13.12	MLS = 1.72 (p = 0.0043)	110 AGRE families
	Broad ASD diagnosis	8q24.13	MLS = 1.66 (p = 0.005)	110 AGRE families
	Broad ASD diagnosis	16p13.12	MLS = 1.46 (p = 0.0081)	110 AGRE families
	Narrow ASD diagnosis	19p13.12	MLS = 2.53 (p = 0.00061)	72 AGRE families
	Narrow ASD diagnosis	Xq25	X-MLS = 2.67 (p = 0.0018)	72 AGRE families
	Narrow ASD diagnosis	16p13.12	MLS = 1.93 (p = 0.0026)	72 AGRE families
	Narrow ASD diagnosis	5p15.33	MLS = 1.63 (p = 0.0054)	72 AGRE families
	Narrow ASD diagnosis	5p13.1	MLS = 1.41 (p = 0.0092)	72 AGRE families
[12]	Autism diagnosis and phrase speech delay	2q31.3-q32.1	Z = 3.32 (P = 0.00038)	49 AGRE/Seaver center families
[6]	ASD diagnosis	2q24-2q31	MLS = 3.74	152 IMGSAC families
	Strict Autism diagnosis	2q24-2q31	MLS = 4.80 (P = 0.00002)	127 IMGSAC families
	ASD diagnosis	7q22-31	MLS = 3.20 (P = 0.0006)	152 IMGSAC families
	ASD diagnosis	16p13	MLS = 2.93	152 IMGSAC families
[13]	ASD diagnosis	3q25-27	Z_{max} = 4.31	28 Finnish families

Table S1. ***Continue***

[14]	Broad ASD diagnosis	2q33.1	MLS = 1.12	82 families
	ASD and phrase speech delay>36 Mo	2q33.1	MLS = 2.86	45 families
	ASD and phrase speech delay>36 Mo	2q33.1	MLS = 1.58	45 families
[15]	ASD and stereotyped patterns/repetitive behaviors on ADI-R	15q11-q13	Dom LOD = 4.71, Rec LOD = 3.83	23 families
	ASD diagnosis	15q11-q13	Dom LOD = 1.40, Rec LOD = 1.07	81 families
[8]	Broad ASD diagnosis	5p13-5p14	MLS = 2.54 (p = 0.00059)	345 AGRE families
	Broad ASD diagnosis	17q11.2	MLS = 2.83 (p = 0.00029)	345 AGRE families
	Broad ASD diagnosis	11p13-11p11.2	MLS = 2.24 (p = 0.0012)	345 AGRE families
[16]	ASD diagnosis, male only	17q11	MLS = 4.3 (P = 0.008)	257 AGRE families, 148 male only
[17]	ASD diagnosis	3q25-27	NPL = 3.5 (p = 0.0003)	A large Utah pedigree
[18]	ASD diagnosis, no affected females	17q11-17q21	LOD = 4.1 (p = 0.00008)	91 AGRE families, 48 male only
[19]	Age at first words	9q33-9q34	Z=3.5 (P = 0.0002)	222 CPEA families
	Strict Autism diagnosis	7q32.1-32.2	P = 0.0006	169 families
	Male only, broad diagnosis	11q13.4	P = 0.0009	148 families
	Female containing	4q24	P = 0.002	74 families
[20]	Social Responsiveness Score	11p12-11p13	Zmax = 3.2 (P = 0.0007)	99 AGRE families
[21]	ASD diagnosis	11p12	Z = 3.6	1181 AGP families
[22]	ASD diagnosis	12q13.13-q15	HLOD = 3.02	26 extended families
	ASD diagnosis, male only affected families	12q13.13-q15	Rec HLOD = 4.51 (P = 0.001)	17 extended families
[23]	ASD diagnosis	1q23	p = 0.00082	An extended-Finnish family
		15q11-q13	P = 0.00084	An extended-Finnish family
		19p13.3	P = 0.000078	An extended-Finnish family
[30]	ASD diagnosis	20p13	LOD=3.81	878 families
		6q27	LOD=2.94	878 families
[24]	ASD diagnosis, high risk families	Xp22.11-21.2	max LOD=2.01, dom model	86 pedigrees
[25]	ASD diagnosis and IQ	10p12	p=0.001	287 multiplex families
		16q23	p=0.015	287 multiplex families
		2p21	p=0.03	287 multiplex families

METHODS-Supplemental information

ADI-R Subtyping

Phenotypic subtyping of the probands was assigned using previously performed ADI-R cluster analyses methods [27]. Briefly, this involved K-means cluster analyses (K = 4) to divide the initial 1954 AGRE probands into four subgroups based upon severity scores on 123 items probed by the ADI-R assessment measure. Four subgroups were determined to be the optimal number for the ASD population examined based on prior Figure of Merit analysis of the ADI-R dataset as described [27]. Unsupervised principal components analysis was also used to confirm the phenotypic similarity of individual cases within the four subgroups based on their respective aggregate ADI-R severity profiles across all selected items. All analyses were performed using the Multi-experiment Viewer (MeV) software developed by Quackenbush and colleagues [28].

Linkage Analysis

Linkage analysis was performed using the described stratification protocol which resulted in 16 subgroup-specific datasets. Two-point non-parametric linkage (NPL) was performed using MERLIN version 1.1.2, [29] and Whittemore and Halpern NPL LOD scores were calculated using the Kong and Cox linear model. Linkage analysis of chromosome X was done using MINX, an X-specific version linkage tool available as part of the MERLIN software. High SNP density can lead to an increased likelihood that the SNPs could be in linkage disequilibrium (LD), and the failure to evaluate for marker-marker LD can cause a false inflation of LOD scores [30]. To address this concern, we used two independent SNP cohorts and focused on regions that generated suggestive linkage using both of these cohorts. Furthermore, the SNP cohort 2 contains a pruned set of high quality polymorphic markers which have been adjusted for LD by removing nearby correlated markers with $r^2>0.1$, as previously described [31].

Permutation for Linkage Analysis

To assess how often a similar significant linkage result (i.e., max LOD scores) might arise by chance, we used the simulation function in MERLIN. A total of 100 simulated genotype data for autosomal SNPs (i.e., 16,303 markers in the SNP dataset-2) were generated for ALL, the original non-stratified group (referring to this simulated dataset as Sim100.ALL). The pedigree structures and affected status were preserved in the simulated data. The same ADI-R related stratification was then applied on the Sim100.ALL pedigree files to generate 100 simulated datasets for each of the 16 subsets. Genome-wide linkage was performed on Sim100.ALL and the generated subsets (e.g., Sim100.G1, Sim100.G1s, etc). The highest LOD score was recorded from Sim100.ALL and subset-simulated analyses. The maximum LOD for each simulated dataset (across Sim100.ALL and resultant simulated subsets) were ranked to calculate study-wide significant levels (using $p<0.05$ as threshold) for the observed LOD scores in the actual datasets. See **Figure S2** and **Tables S9A-B (**in **Files S3** and **S4)** for detail on the applied workflow for permutation analysis and the generated data, respectively. Throughout this paper, we refer to LOD scores >3.0 as "suggestive" linked regions if they did not pass the permutation test.

Association Analysis

The transmission disequilibrium test (TDT) [32] was used for association analysis because the TDT is not biased by population stratification. SNPs passing quality control from the Weiss *et al*. [31] paper were used for the TDT association analysis. Only one affected subject per family was included in the TDT analysis to reduce finding associations as a reflection of linkage profile in the pedigrees showing significant linkage peaks. Detailed description of data cleaning and filtering has been discussed elsewhere [31]. Association analysis was performed using PLINK [33].

Visualization of LOD Scores and Cluster Analyses of Linkage Data across Subtypes

MeV software [28] was used to permit visual comparison of suggestive linkage regions (using the LOD scores) across the 16 subgroups in comparison to that of the undivided ALL group. Unsupervised hierarchical clustering and principal components analysis of linked loci with LOD scores ≥ 2 in at least one of the subgroups were also conducted using MeV to demonstrate the subgroup-dependent linkage "hotspots" in a more unbiased manner.

RESULTS-Supplemental information

Table S2. The number of multiplex families, in each subgroup, without (n1) and with (n2) BroadSpectrum subjects.

Group	n1	n2 *(w%)*
**ALL**[a]	337	392 *(76%)*
**G1**	194	232 *(83%)*
**G1s**	41	63 *(97%)*
**G1M**	25	39 *(95%)*
**G1Fc***	15	15 *(100%)*
**G2**	157	185 *(84%)*
**G2s**	19	25 *(88%)*
**G2M**	12	16 *(88%)*
**G2Fc***	8	8 *(88%)*
**G3**	138	159 *(80%)*
**G3s**	31	35 *(85%)*
**G3M**	22	25 *(84%)*
**G3Fc***	9	9 *(88%)*
**G4**	116	126 *(76%)*
**G4s**	13	16 *(71%)*
**G4M**	7	8 *(63%)*
**G4Fc***	6	6 *(67%)*

*The number of families did not change in the Fc subsets, after including BroadSpectrum subjects; Fc=female-containing family

[a]The original unstratified cohort

The respective sizes of the resulting 16 subgroups are shown. Due to the existing intra-family heterogeneity, some families were included in more than one phenotypic subgroup. Therefore, the sum of family numbers in subgroups exceeds the numbers listed for the original cohort (ALL).

w%= The prevalence of the common race (i.e., white) in each subgroups

Table S3. Overlap between the subgroups at the G level.

	Affected subjects per ADI-R related subgroups (%)			
**ADI-R subtyping**	**G1**	**G2**	**G3**	**G4**
g1 subject	**59%**	17%	14%	10%
g2 subject	22%	**52%**	14%	12%
g3 subject	20%	14%	**54%**	10%
g4 subject	19%	17%	14%	**50%**

As expected, in each G level subgroup the highest % of the included autistic subjects (≥50%) belong to the initial subgroup with the respective ADI-R determined sub-phenotype, shown in gray-shaded cells with bold font. To distinguish resultant subsets from the ADI-R clusters, the four ADI-R subtypes are labeled as g1, g2, g3, and g4.

Table S5. Chromosomal locations of the positive linked loci (LOD≥ 2) and their associated genes per subgroups. The number of families examined to identify suggestive linked loci are listed.

**Subgroup (# families)**	**Chromosomal location (LOD)**	**Genes associated with SNPs with LOD ≥ 2**
**ALL (392)**	17p11.2 (2.06-2.95)	ALDH3A1, C17ORF108, C17ORF63, EVPLL, FLCN, KCNJ12, KSR1, LGALS9, MYO1D, NF1, NOS2, PIPOX, PRPSAP2, SLC47A1, SPACA3, TBC1D29, TMEM97, ULK2
**G1 (232)**	13q14.1-q14.3 (2.04-3.39)	CAB39L, CPB2, DHRS12, DLEU7, FAM10A4, FAM124A, FLJ3707, GUCY1B2, HTR2A, LCP1, LRCH1, PHF11, VPS36
G1	13q21.2-q21.33 (3.4-4.37)	ATXN8OS, DACH1, DIAPH3, KLHL1, PCDH9
G1	13q22.1-q22.2 (2-2.53)	KLF12, LMO7, LOC647288, PIBF1, TBC1D4, UCHL3
**G1s (63)**	22q11.1-q11.23 (2.15-4.43)	BCR, BID, CABIN1, CECR1, CECR2, CRYBB3, CYTSA, DGCR14, FLJ41941, GSTTP2, IL17RA, IGLL1, KIAA1671, LOC91316, MAPK1, MED15, MICAL3, MIF, P2RX6, PI4KA, RAB36, SCARF2, SGSM1, SLC2A11, SMARCB1, TBX1, TXNRD2, UFD1L, USP18
**G1Fc (15)**	22q11.1-q11.23 (2.09-2.54)	BCR, DGCR14, MAPK1, MED15, MICAL3, P2RX6, PI4KA, RAB36, SCARF2, TXNRD2, UFD1L, USP18
**G1M (39)**	3q28 (2.02-2.1)	IL1RAP
G1M	15q25.1-q25.3 (2.03-2.52)	ACSBG1, ADAMTSL3, C15ORF37, CPEB1, DNAJA4, FAM154B, HOMER2, IDH3A, KLHL25, NCRNA00052, NMB, NTRK3, PDE8A, RASGRF1, SH3GL3, TBC1D2B, WDR61
**G2 (185)**	4q13.1-q13.2 (2.02-2.47)	EPHA5, STAP1, TECRL
G2	4q22.3 (2.02-2.13)	UNC5C
G2	4q23 (2-2.17)	ADH1B, C4ORF37, EIF4E, RAP1GDS1, TSPAN5
G2	10q26.3 (2.01-2.05)	LRRC27, STK32C
G2	11q12.3 (2.03)	AHNAK

Table S5. ***Continue***

**G2M (16)**	6q27 (2.07-2.27)	RPS6KA2
G2M	12p13.2-p13.31 (2.07-2.15)	A2ML1, CD69, CLEC1B, CLEC4D, PZP, RIMKLB
**G3s (35)**	12p12.3 (2.02-2.15)	PIK3C2G
**G3M (25)**	4p15.33 (2-2.19)	HS3ST1
G3M	4p16.1 (2.28-2.39)	CLNK
G3M	15q24.1-q24.3 (2-2.2)	C15ORF27, LINGO1, MPI, NRG4, ODF3L1, SIN3A, SCAPER
G3M	15q25.1-q25.3 (2.02-2.2)	ACSBG1, BCL2A1, C15ORF37, DNAJA4, IDH3A, RASGRF1, TBC1D2B, WDR61
**G4 (126)**	5p13.3 (2-2.01)	ADAMTS12
**G4s (16)**	3p14.1 (2.11-2.54)	LRIG1, MAGI1, MIR548A2
G4s	5p15.2-p15.31 (2-2.05)	LOC285692, SEMA5A
G4s	6q25.1 (2-2.03)	PLEKGH1, UST, ZC3H12D
G4s	10q23.1-q23.33 (2-2.05)	ANKRD22, ATAD1, BMPR1A, EXOC6, GHITM, GRID1, IDE, IFIT2, LDB3, LIPA, LIPJ, LIPM, LOC10018894, MYOF, PANK1, SLC16A12, STAMBPL1
G4s	11p14.3 (2-2.11)	GAS2, SLC17A6
G4s	11p15.1 (2)	ABCC8, GTF2H1, NAV2, NELL1, PLEKHA7, PRMT3, PTPN5, SAA4, SERGEF, SLC6A5, TPH1, UEVLD, USH1C
G4s	12q15 (2.07)	PTPRR
G4s	12q21.1-q21.2 (2-3.56)	CSRP2, E2F7, KCNC2, LGR5, LOC283392, NAP1L1, NAV3, TPH2, TRHDE, TSPAN8, ZDHHC17

Table S6. TDT result for two previously associated SNPs at chromosome 5p*.

dataset (no of pedigrees)	SNP	A1	A2	T	U	OR	P
G1.2Fc (23)	rs10513025[a]	G	A	0	4	0	0.0455
G1.2Fc (23)	rs4307059[b]	G	A	4	14	0.2857	0.01842
All.Fc (166)	rs10513025[a]	G	A	7	10	0.7	0.4669
All.Fc (166)	rs4307059[b]	G	A	56	67	0.8358	0.3213

Transmitted (T) and untransmitted (U) counts and odds ratios (OR) for the minor allele (A1) are shown for each SNP

[a] the most significant SNP reported by Weiss *et al*. (31)

[b] the most significant SNP reported by Wang *et al*. (26)

*Our study found a suggestive linkage at the 5p locus for the combined G1.2Fc group (23 pedigrees). To assess associations with the previously reported SNPs for this chromosomal region, TDT association analyses were performed on this ADI-R stratified group compared with All.Fc (166 pedigrees), which includes all female-containing pedigrees. Only one affected sibling per each pedigree was included for the TDT analysis to avoid detecting associations because of linked SNPs.

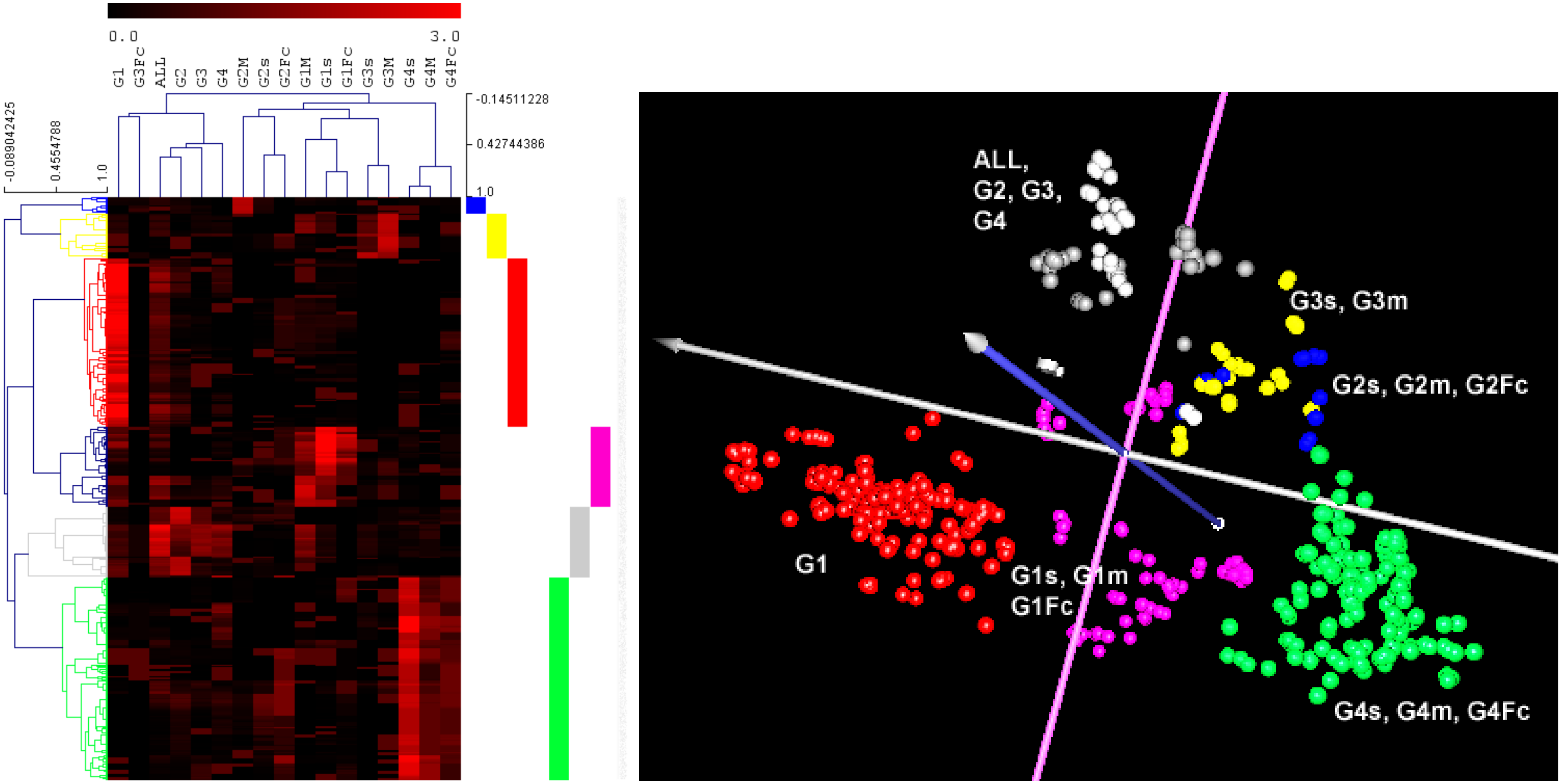

1A **1B**

Figure S1. Hierarchical clustering and principal components analyses. S1A (left) shows the results of unsupervised hierarchical clustering of subgroups and loci with LOD ≥ 2 in at least one ASD subgroup. Each column represents a subgroup and each row represents a SNP. The length of the branches along both axes is inversely related to the correlation between the subgroups (columns) and loci (rows) as determined by the Pearson coefficient (scales along both axes). **S1B** (right) shows the results of principal components analyses of the loci, wherein the color corresponds to the major branches along the SNP axis in **Figure S1A**. Magenta is used for stratified subgroups G1s, G1M, and G1Fc, while red is used for the G1 group.

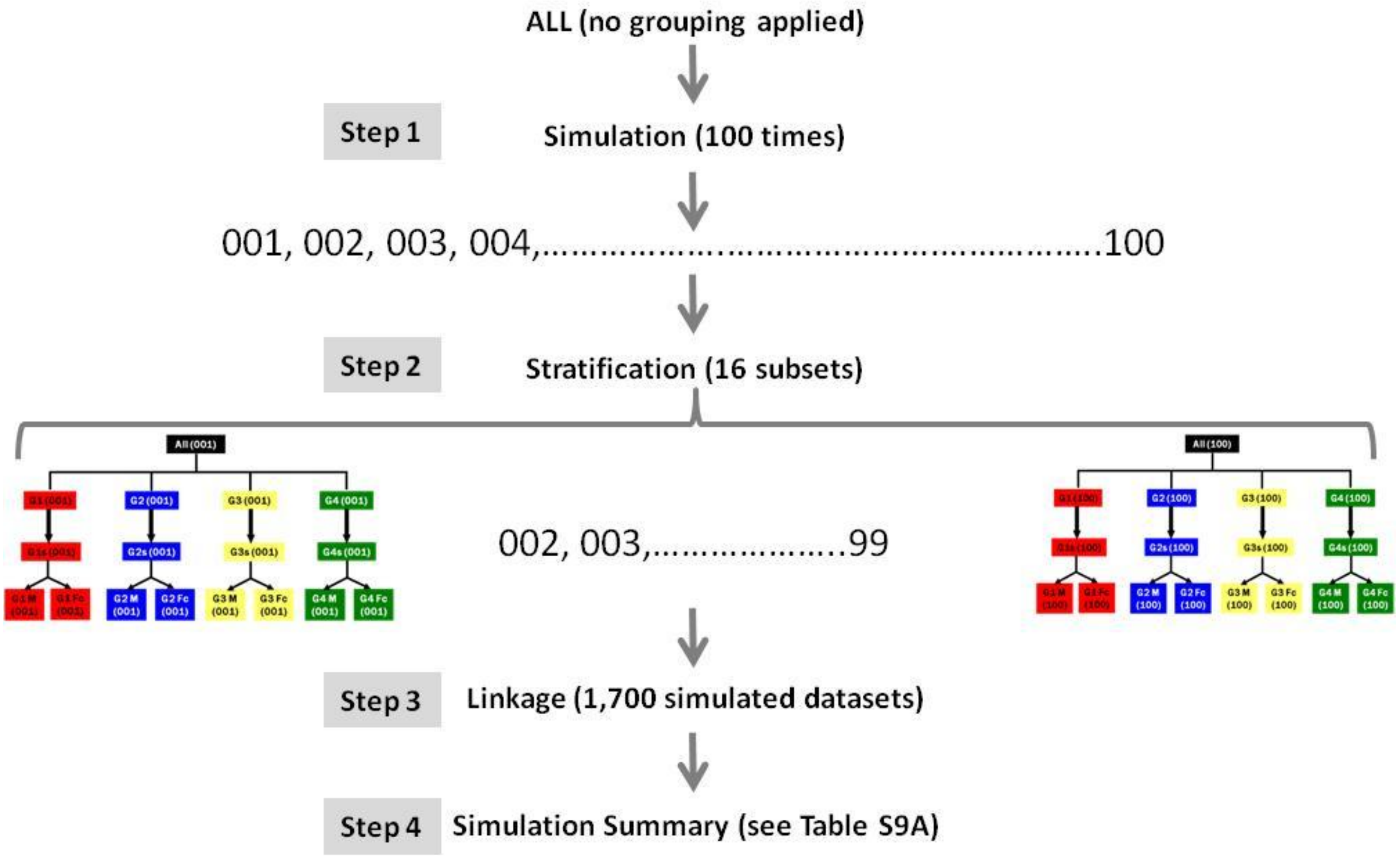

Figure S2. Workflow describing the applied permutation analysis. Simulation analysis involved the following steps. **Step 1:** performed 100 simulations on the main group (i.e., ALL); **Step 2:** utilized the same stratification method as what was applied on the actual dataset (see **Figure 1**) to generate the same 16 subgroups, resulting in 1,700 simulated files for genome-wide scans; **Step 3:** performed genome-wide scan on 1,700 simulated files; and **Step 4:** evaluated LOD scores for calculating empirical p values [see **Table S9A** (**File S3**) for data]. Furthermore, similarly 100 simulated files were generated for the three combined groups that we have tested (e.g., G1.2Fc, etc), resulting in 300 more simulated files. Therefore, overall a total of 2000 genome-wide scans were generated and analyzed for permutation analysis [see **Table S9B** (**File S4**)].

DISCUSSIONS-Supplemental information

Additional Potential Candidate Genes in the Linked Regions

Supplementary **Tables S7 and S8** list the genes associated with the SNPs with the highest LOD scores in 13q21 and 22q11 regions, respectively. One of the genes in the 13q21 linked region is *PCDH9*, a neuronal protocadherin that is a component of synaptic complexes. *PCDH9* was previously found to be associated with ASD by CNV analyses [34]. Another potentially relevant gene in this region is *KLHL1*, which is associated with gait disturbance [35], a motor phenotype affecting some individuals with ASD [36]. An antisense transcript to *KLHL1* (*ATXN8OS* or *KLHL1AS*) is also within the linked region. Expansion of unstable trinucleotide repeat tracts in *ATXN8OS* has been associated with spinocerebellar ataxia type 8, a late-onset progressive neurodegenerative disorder also featuring severe gait, speech and sensory loss. Long repeat tracts of this transcript have been also reported in subjects with schizophrenia and bipolar disorder [37]. Down regulating *KLHL1* expression through an antisense mechanism has been shown as a potential way that repeat expansions in this non protein-coding RNA may lead to neuropathogenesis [38].

With respect to candidate genes on 22q11, *MAPK1*, *MICAL3*, and *USP18* fall in the linkage interval (see **Table S8**). While *MAPK1* is a critical component of many signaling pathways, including MTOR signaling which is strongly implicated in ASD and related disorders, *MICAL3* is specifically involved in semaphorin-Plexin A signaling in motor neurons [39]. *USP18*, on the other hand, is a ubiquitin-specific protease that plays a role in interferon response to viral infection of brain cells [40] and in innate immunity [41], which has been suggested to contribute to the neuropathology of ASD [42,43].

Table S7. List of the genes associated with the SNPs with the highest LOD scores in 13q21 (G1 group).

Gene	LOD	SNP	Chromosomal location	SNP's position in gene
PCDH9	4.37	rs4142274	13q21.32	Intron
PCDH9	4.36	rs4883796	13q21.32	Intron
PCDH9	4.35	rs9317631	13q21.32	Intron
PCDH9	4.26	rs913493	13q21.32	Intron
PCDH9	4.24	rs2324967	13q21.32	Intron
PCDH9	4.22	rs7324330	13q21.32	Intron
KLHL1	3.98	rs7986686	13q21.33	Intron
PCDH9	3.86	rs11148709	13q21.32	Intron
PCDH9	3.71	rs166500	13q21.32	Intron
KLHL1	3.69	rs4884871	13q21.33	Intron
ATXN8OS	3.66	rs9564649	13q21.33	Promoter
KLHL1	3.66	rs683300	13q21.33	Intron
ATXN8OS	3.65	rs9599553	13q21.33	Intron
PCDH9	3.64	rs9540711	13q21.32	Intron
ATXN8OS	3.64	rs9529683	13q21.33	Downstream
DIAPH3	3.11	rs1337645	13q21.2	Intron
DIAPH3	3.11	rs342594	13q21.2	Intron
DACH1	2.75	rs966168	13q21.33	Intron

Table S8. List of the genes associated with the SNPs with the highest LOD scores in 22q11 (G1s group).

Gene	LOD	SNP	Chromosomal location	SNP's position in gene
MAPK1	4.43	rs2283792	22q11.21	Intron
FLJ41941	4.41	rs462904	22q11.21	Downstream
MICAL3	4.4	rs452579	22q11.21	Intron
MICAL3	4.38	rs424765	22q11.21	Intron
MICAL3	4.36	rs9604803	22q11.21	Intron
USP18	4.28	rs2252257	22q11.21	Intron (boundary)
BID	3.99	rs181408	22q11.21	Intron
P2RX6	3.86	rs8141816	22q11.21	Intron
CECR2	3.72	rs1296795	22q11.21	Intron (boundary)
PI4KA	3.59	rs165924	22q11.21	Intron (boundary)
RAB36	3.59	rs5751592	22q11.22	Intron
DGCR14	3.58	rs16983371	22q11.21	Intron
BCR	3.58	rs2071436	22q11.23	Intron
BCR	3.53	rs7288846	22q11.23	Intron
CECR2	3.45	rs2518768	22q11.21	Intron
UFD1L	3.44	rs756658	22q11.21	Intron
TBX1	2.96	rs5748427	22q11.21	Downstream
TXNRD2	2.93	rs2073750	22q11.21	Intron
TXNRD2	2.89	rs5993875	22q11.21	Intron
IL17RA	2.78	rs2241049	22q11.1	Intron
MED15	2.77	rs7292126	22q11.21	Intron
SCARF2	2.76	rs882745	22q11.21	Intron (boundary)
CECR1	2.73	rs1076106	22q11.1	Intron
IGLL1	2.73	rs7287616	22q11.23	Promoter
LOC91316	2.7	rs738785	22q11.23	Intron
SMARCB1	2.57	rs2267032	22q11.23	Intron
SGSM1	2.38	rs6004307	22q11.23	Intron
SLC2A11	2.31	rs738803	22q11.23	Promoter
MIF	2.25	rs738806	22q11.23	Promoter
GSTTP2	2.18	rs738809	22q11.23	Promoter
KIAA1671	2.18	rs984814	22q11.23	Intron
SGSM1	2.18	rs7287595	22q11.23	Intron
CABIN1	2.17	rs2267064	22q11.23	Intron
CYTSA	2.17	rs2082733	22q11.23	Intron
CRYBB3	2.15	rs1054476	22q11.23	Downstream

REFERENCES-Supplemental information

1. Freitag CM, Staal W, Klauck SM, Duketis E, Waltes R (2010) Genetics of autistic disorders: review and clinical implications. Eur Child Adolesc Psychiatry 19: 169-178.
2. Abrahams BS, Geschwind DH (2008) Advances in autism genetics: on the threshold of a new neurobiology. Nat Rev Genet 9: 341-355.
3. Weiss LA (2009) Autism genetics: emerging data from genome-wide copy-number and single nucleotide polymorphism scans. Expert Rev Mol Diagn 9: 795-803.
4. Craddock N, Lendon C (1999) Chromosome Workshop: chromosomes 11, 14, and 15. Am J Med Genet 88: 244-254.
5. IMGSAC (1998) A full genome screen for autism with evidence for linkage to a region on chromosome 7q. . Hum Mol Genet 7: 571-578.
6. IMGSAC (2001) A genomewide screen for autism: strong evidence for linkage to chromosomes 2q, 7q, and 16p. Am J Hum Genet 69: 570-581.
7. Liu J, Nyholt DR, Magnussen P, Parano E, Pavone P, et al. (2001) A genomewide screen for autism susceptibility loci. Am J Hum Genet 69: 327-340.
8. Yonan AL, Alarcon M, Cheng R, Magnusson PK, Spence SJ, et al. (2003) A genomewide screen of 345 families for autism-susceptibility loci. Am J Hum Genet 73: 886-897.
9. Barrett S, Beck JC, Bernier R, Bisson E, Braun TA, et al. (1999) An autosomal genomic screen for autism. Collaborative linkage study of autism. Am J Med Genet 88: 609-615.
10. Risch N, Spiker D, Lotspeich L, Nouri N, Hinds D, et al. (1999) A genomic screen of autism: evidence for a multilocus etiology. Am J Hum Genet 65: 493-507.
11. Philippe A, Martinez M, Guilloud-Bataille M, Gillberg C, Rastam M, et al. (1999) Genome-wide scan for autism susceptibility genes. Paris Autism Research International Sibpair Study. Hum Mol Genet 8: 805-812.
12. Buxbaum JD, Silverman JM, Smith CJ, Kilifarski M, Reichert J, et al. (2001) Evidence for a susceptibility gene for autism on chromosome 2 and for genetic heterogeneity. Am J Hum Genet 68: 1514-1520.
13. Auranen M, Vanhala R, Varilo T, Ayers K, Kempas E, et al. (2002) A genomewide screen for autism-spectrum disorders: evidence for a major susceptibility locus on chromosome 3q25-27. Am J Hum Genet 71: 777-790.
14. Shao Y, Raiford KL, Wolpert CM, Cope HA, Ravan SA, et al. (2002) Phenotypic homogeneity provides increased support for linkage on chromosome 2 in autistic disorder. Am J Hum Genet 70: 1058-1061.
15. Shao Y, Cuccaro ML, Hauser ER, Raiford KL, Menold MM, et al. (2003) Fine mapping of autistic disorder to chromosome 15q11-q13 by use of phenotypic subtypes. Am J Hum Genet 72: 539-548.
16. Stone JL, Merriman B, Cantor RM, Yonan AL, Gilliam TC, et al. (2004) Evidence for sex-specific risk alleles in autism spectrum disorder. Am J Hum Genet 75: 1117-1123.

17. Coon H, Matsunami N, Stevens J, Miller J, Pingree C, et al. (2005) Evidence for linkage on chromosome 3q25-27 in a large autism extended pedigree. Hum Hered 60: 220-226.
18. Cantor RM, Kono N, Duvall JA, Alvarez-Retuerto A, Stone JL, et al. (2005) Replication of autism linkage: fine-mapping peak at 17q21. Am J Hum Genet 76: 1050-1056.
19. Schellenberg GD, Dawson G, Sung YJ, Estes A, Munson J, et al. (2006) Evidence for multiple loci from a genome scan of autism kindreds. Mol Psychiatry 11: 1049-1060, 1979.
20. Duvall JA, Lu A, Cantor RM, Todd RD, Constantino JN, et al. (2007) A quantitative trait locus analysis of social responsiveness in multiplex autism families. Am J Psychiatry 164: 656-662.
21. Szatmari P, Paterson AD, Zwaigenbaum L, Roberts W, Brian J, et al. (2007) Mapping autism risk loci using genetic linkage and chromosomal rearrangements. Nat Genet 39: 319-328.
22. Ma DQ, Cuccaro ML, Jaworski JM, Haynes CS, Stephan DA, et al. (2007) Dissecting the locus heterogeneity of autism: significant linkage to chromosome 12q14. Mol Psychiatry 12: 376-384.
23. Kilpinen H, Ylisaukko-oja T, Rehnstrom K, Gaal E, Turunen JA, et al. (2009) Linkage and linkage disequilibrium scan for autism loci in an extended pedigree from Finland. Hum Mol Genet 18: 2912-2921.
24. Allen-Brady K, Cannon D, Robison R, McMahon WM, Coon H (2010) A unified theory of autism revisited: linkage evidence points to chromosome X using a high-risk subset of AGRE families. Autism Res 3: 47-52.
25. Chapman NH, Estes A, Munson J, Bernier R, Webb SJ, et al. (2011) Genome-scan for IQ discrepancy in autism: evidence for loci on chromosomes 10 and 16. Hum Genet 129: 59-70.
26. Wang K, Zhang H, Ma D, Bucan M, Glessner JT, et al. (2009) Common genetic variants on 5p14.1 associate with autism spectrum disorders. Nature 459: 528-533.
27. Hu VW, Steinberg ME (2009) Novel clustering of items from the Autism Diagnostic Interview-Revised to define phenotypes within autism spectrum disorders. Autism Res 2: 67-77.
28. Saeed AI, Sharov V, White J, Li J, Liang W, et al. (2003) TM4: a free, open-source system for microarray data management and analysis. Biotechniques 34: 374-378.
29. Abecasis GR, Cherny SS, Cookson WO, Cardon LR (2002) Merlin--rapid analysis of dense genetic maps using sparse gene flow trees. Nat Genet 30: 97-101.
30. Abecasis GR, Wigginton JE (2005) Handling marker-marker linkage disequilibrium: pedigree analysis with clustered markers. Am J Hum Genet 77: 754-767.
31. Weiss LA, Arking DE, Daly MJ, Chakravarti A (2009) A genome-wide linkage and association scan reveals novel loci for autism. Nature 461: 802-808.
32. Spielman RS, McGinnis RE, Ewens WJ (1993) Transmission test for linkage disequilibrium: the insulin gene region and insulin-dependent diabetes mellitus (IDDM). Am J Hum Genet 52: 506-516.

33. Purcell S, Neale B, Todd-Brown K, Thomas L, Ferreira MA, et al. (2007) PLINK: a tool set for whole-genome association and population-based linkage analyses. Am J Hum Genet 81: 559-575.
34. Marshall CR, Noor A, Vincent JB, Lionel AC, Feuk L, et al. (2008) Structural variation of chromosomes in autism spectrum disorder. Am J Hum Genet 82: 477-488.
35. He Y, Zu T, Benzow KA, Orr HT, Clark HB, et al. (2006) Targeted deletion of a single Sca8 ataxia locus allele in mice causes abnormal gait, progressive loss of motor coordination, and Purkinje cell dendritic deficits. J Neurosci 26: 9975-9982.
36. Maski KP, Jeste SS, Spence SJ (2011) Common neurological co-morbidities in autism spectrum disorders. Curr Opin Pediatr 23: 609-615.
37. Vincent JB, Yuan QP, Schalling M, Adolfsson R, Azevedo MH, et al. (2000) Long repeat tracts at SCA8 in major psychosis. Am J Med Genet 96: 873-876.
38. Chen WL, Lin JW, Huang HJ, Wang SM, Su MT, et al. (2008) SCA8 mRNA expression suggests an antisense regulation of KLHL1 and correlates to SCA8 pathology. Brain Res 1233: 176-184.
39. Bron R, Vermeren M, Kokot N, Andrews W, Little GE, et al. (2007) Boundary cap cells constrain spinal motor neuron somal migration at motor exit points by a semaphorin-plexin mechanism. Neural Dev 2: 21.
40. van den Pol AN, Robek MD, Ghosh PK, Ozduman K, Bandi P, et al. (2007) Cytomegalovirus induces interferon-stimulated gene expression and is attenuated by interferon in the developing brain. J Virol 81: 332-348.
41. Ritchie KJ, Hahn CS, Kim KI, Yan M, Rosario D, et al. (2004) Role of ISG15 protease UBP43 (USP18) in innate immunity to viral infection. Nat Med 10: 1374-1378.
42. Vargas DL, Nascimbene C, Krishnan C, Zimmerman AW, Pardo CA (2005) Neuroglial activation and neuroinflammation in the brain of patients with autism. Ann Neurol 57: 67-81.
43. Pardo CA, Vargas DL, Zimmerman AW (2005) Immunity, neuroglia and neuroinflammation in autism. Int Rev Psychiatry 17: 485-495.
